# Recent Progress on Anti-Humidity Strategies of Chemiresistive Gas Sensors

**DOI:** 10.3390/ma15248728

**Published:** 2022-12-07

**Authors:** Yanjie Wang, Yong Zhou

**Affiliations:** Key Laboratory of Optoelectronic Technology and System of Ministry of Education, College of Optoelectronic Engineering, Chongqing University, Chongqing 400044, China

**Keywords:** anti-humidity chemiresistive gas sensor, surface engineering, physical isolation, working parameter modulation, algorism compensation, novel material development

## Abstract

In recent decades, chemiresistive gas sensors (CGS) have been widely studied due to their unique advantages of expedient miniaturization, simple fabrication, easy operation, and low cost. As one ubiquitous interference factor, humidity dramatically affects the performance of CGS, which has been neglected for a long time. With the rapid development of technologies based on gas sensors, including the internet of things (IoT), healthcare, environment monitoring, and food quality assessing, the humidity interference on gas sensors has been attracting increasing attention. Inspiringly, various anti-humidity strategies have been proposed to alleviate the humidity interference in this field; however, comprehensive summaries of these strategies are rarely reported. Therefore, this review aims to summarize the latest research advances on humidity-independent CGS. First, we discussed the humidity interference mechanism on gas sensors. Then, the anti-humidity strategies mainly including surface engineering, physical isolation, working parameters modulation, humidity compensation, and developing novel gas-sensing materials were successively introduced in detail. Finally, challenges and perspectives of improving the humidity tolerance of gas sensors were proposed for future research.

## 1. Introduction

In the last decades, gas-detection technologies have been widely employed in various application scenarios, such as the internet of things (IoT), healthcare, industrial and agricultural safety, environment monitoring, and food quality assessment [1,2,3,4,5]. Among these technologies, chemiresistive gas sensors prevail over their optical, mass-sensitive, and electrochemical counterparts due to their unique advantages of small size, convenient fabrication, simple operation, and high cost-efficiency [6,7,8]. For a long time, the “4S” standard, namely sensitivity, speed, stability, and selectivity, dominates the design criteria of gas sensors [9,10]. Additionally, the process of sensor design always ends in satisfactory results when achieving these goals, including high sensitivity, fast response/recovery speed, strong stability, and exclusive selectivity under the laboratory environment. However, during the practical applications of CGS, ambient environmental parameters, including ambient temperature, relative humidity, and other interference gases significantly impact the gas-sensing performance [11,12,13]. Of these parameters, ambient humidity is one ubiquitous influence factor, especially for the breath analysis in the presence of more than 80% RH and environment monitoring, which features high-humidity backgrounds [14,15].

In regard to CGS, the concentration of target gas is closely related to the electrical signal (e.g., resistance and current) of the sensors upon molecular adsorption. As the active centers of CGS, various sensitive materials have been investigated, such as polymers, metal oxides (ZnO, SnO_2_, In_2_O_3_, etc.), and two-dimensional layered nanomaterials (graphene, transition metal dichalcogenides (TMDs), black phosphorus, etc.) [16,17]. However, a large number studies have verified that most of these materials are very susceptible to water vapor [18,19,20]. When the humidity within the operating environment changes, the baseline resistance of CGS and response to target gas substantially fluctuated, which seriously deteriorated the measurement accuracy [21,22]. Therefore, it is essential to overcome this notable setback to facilitate further applications.

Recently, humidity interference has been causing increasing attention in the design of gas sensors [23]. Various strategies have been proposed to improve the humidity independence of gas sensors [24,25]. For instance, Li et al. [26] fabricated an NH_3_ gas sensor by the thermal evaporation of CuBr on a polyimide substrate and coating of CeO_2_ by electron-beam evaporation. The CuBr sensor coated with the 100-nm-thick CeO_2_ overlayers showed nearly unvaried sensing response toward NH_3_ over a wide humidity range of 0–80% RH, surpassing pure CuBr counterpart. The excellent anti-humidity property provided the CeO_2_-covered CuBr with the ability to recognize the ppb level NH_3_ in human breath for helicobacter pylori diagnosis. Xie et al. [27] utilized NiO to decorate In_2_O_3_ for H_2_S sensing. Almost constant dynamic response curves of the NiO-In_2_O_3_ sensor were respectively obtained at 25% and 75% RH, indicating the remarkable moisture resistance. Wang et al. [28] utilized carbon layers to functionalize WO_3_-W_18_O_49_-PdO composites for anti-humidity triethylamine (TEA) detection. Additionally, the modified sensor exhibited better humidity endurance and a lower detection limit of 50 ppb compared with the unmodified counterparts. Nevertheless, to date, scarce articles have focused on the recent development in anti-humidity strategies for gas sensors. For this reason, in this contribution, we initially discussed the mechanism of humidity interference and the evaluation criterion of the humidity resistance for CGS. Then, diverse methods aiming to improve the anti-humidity features of CGS were categorized and discussed. Finally, a conclusion of all these strategies was presented in terms of advantages, challenges, and the prospects of future work.

## 2. Humidity Interference Mechanism

Water vapor, existing everywhere on the earth, is one notable interference factor for various technologies [29]. Relative humidity (RH) and absolute humidity (AH) are common parameters to estimate the amount of water vapor in the air. Relative humidity, typically expressed as a percentage, is defined as the ratio of the water vapor pressure in air to saturation vapor pressure at the same temperature. Whereas, absolute humidity (AH) describes the concentration of water vapor in the air regardless of temperature with a unit of g/m^3^.

Metal oxide semiconductors (MOS) are popular sensing materials for gas detection, due to their low cost, high sensitivity, and good stability [30,31]. The widely accepted sensing mechanism of gas sensors based on MOS could be closely related to the adsorption of oxygen species in the form of O^2−^, O^−^, and $O_{2}^{-}$ on the surface of MOS [32,33]. These oxygen species originated from ambient oxygen molecules, which serve as the receptor in air to capture electrons from the conduction band of MOS. When MOS sensors are exposed to target gas, the target gas molecules would react with these oxygen species accompanied with releasing or further capturing electrons, thus periodically modulating the resistance or current of sensors. However, when sensors based on MOS are operated in the ambient environment, water vapor could competitively adsorb on the surface of MOS as expressed in Equation (1) [34]:

(1) $$H_{2}O+2M_{m}+O_{\mathrm{ad}}^{-}=2\;(M_{m}^{+}\;-\mathrm{OH})+e^{-}+S$$

where M denotes metal elements in metal oxides, such as Sn, Cu, and Ti; M_m_ is the site of M on the surface; $O_{\mathrm{ad}}^{-}$ presents ionosorbed oxygen species; e^−^ is an electron; ($M_{m}^{+}\;$ − OH) presents a terminal hydroxyl group; and *S* denotes a surface site for chemically adsorbing oxygen.

**Figure 1:**
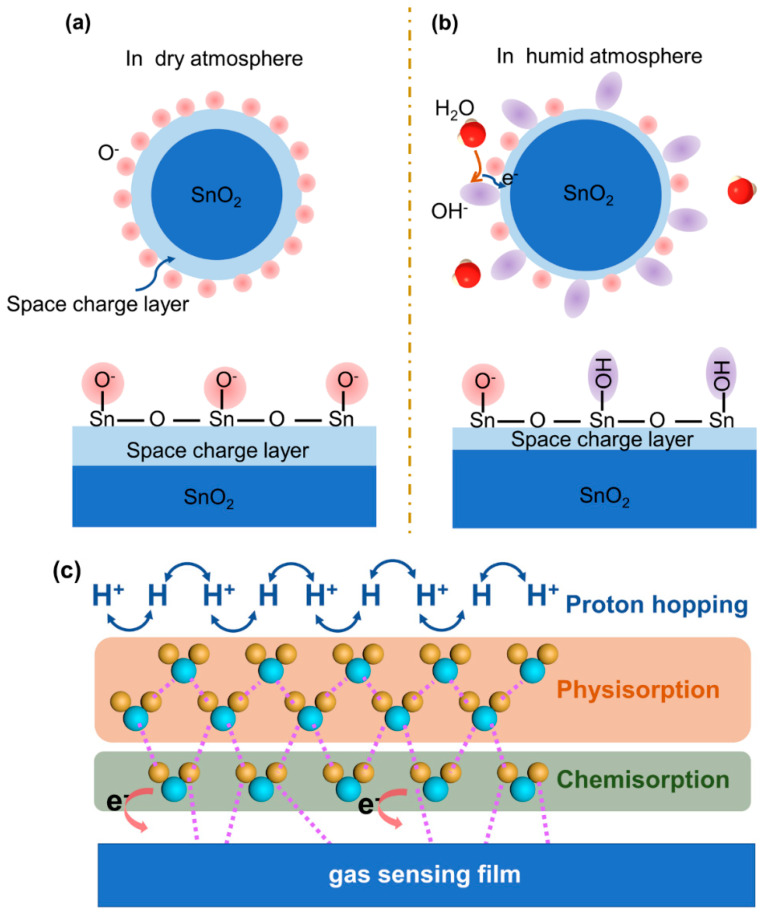
Schematic diagram of gas−sensing model for SnO_2_ in (**a**) dry atmosphere and (**b**) humid atmosphere. (**c**) Schematic illustration of chemisorption and physisorption of water molecules in high-humidity conditions.

Therefore, the chemical adsorption of water molecules could directly change the baseline resistance of the MOS sensor with humid atmospheres. Meanwhile, the concurrent adsorption of water molecules would lead to the decline in response to target gas and thus affect the measurement accuracy. Figure 1a,b schematically exhibited the gas-sensing models of SnO_2_ gas sensors in dry and humid atmospheres, respectively. In addition to MOS, novel two-dimensional (2D) gas materials, carbon-based materials and polymers, are extensively explored for gas sensing [35,36,37]. Different from the gas sensing mechanism of MOS, the target gas would directly react with the adsorption sites on these materials. Analogously, water vapor serving as one reduced gas would adsorb on their surface, thereby altering both base resistance and response to target gases.

The above-mentioned humidity interference mechanism is based on competitive chemisorption, which is also applicable to various interference gases. For typical chemiresistive gas sensors, selectivity is always utilized to evaluate the influence resulting from interference gases. Therefore, it is a feasible method to enhance the selectivity, in order to reduce the influence from common interference gases. In contrast to common interference gases, water molecules present two-step adsorption processes over a wide humidity range, as shown in Figure 1c [38]. As stated above, water molecules were first chemisorbed on the active sites within the sensing film, while electrons were withdrawn from the reducing water. With the increase in humidity, water physisorption was initiated on the chemisorption layer accompanied with the formation of continuous water layers. Then, water molecules under an electrostatic field were ionized to H_3_O^+^, and transferred according to the Grotthuss mechanism (H_2_O + H_3_O^+^ → H_3_O^+^ + H_2_O) [39]. Therefore, the proton hopping markedly promotes the conductivity of the sensing film and thus leads to a considerable decrease in the sensors’ resistance [40]. Due to the unique adsorption mechanism of water molecules, it is difficult to alleviate the humidity effect by simply enhancing selectivity.

## 3. Anti-Humidity Strategies

**Figure 2:**
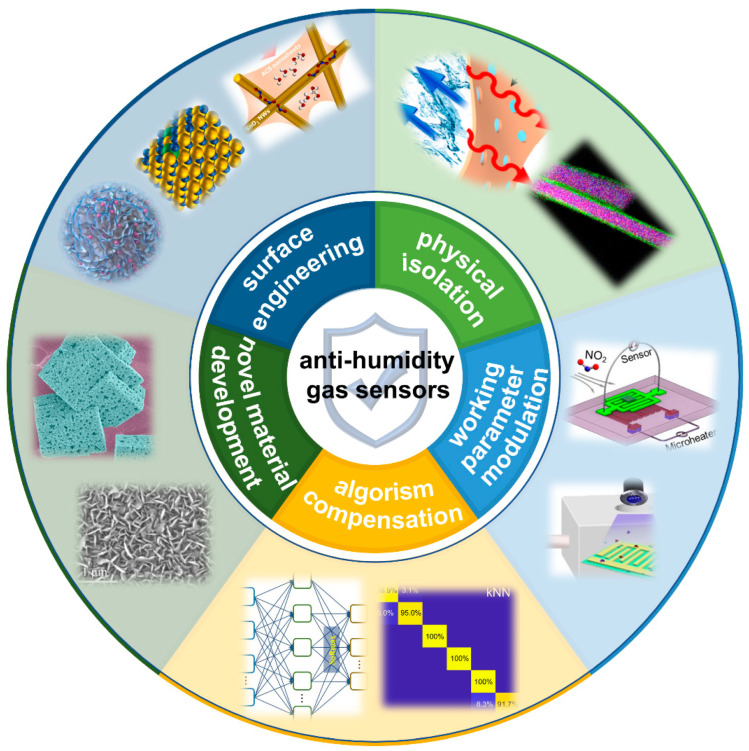
An overview of the anti-humidity strategies of gas sensors, adapted from [41,42,43,44,45,46,47,48,49,50,51].

To suppress the humidity interference on gas detection, various strategies have been proposed, including surface engineering, physical isolation, working parameter modulation, algorism compensation, and novel material development, as shown in Figure 2. These strategies will be successively discussed in the next section in detail.

As stated above, water vapor affects the sensor performance in terms of base resistance and response when humidity changes. Therefore, the coefficient of response variation (CV), baseline resistance drift, baseline resistance ratio, response drift, and response ratio are the widely-accepted criteria to evaluate the performance of anti-humidity effect, and are defined as Equations (2)–(6), respectively:

CV = S_SD_/S_average_ × 100% (2)

where S_SD_ and S_average_ respectively present the standard deviation (SD) and average value of response within different humidity conditions.

Baseline resistance drift (%) = (R_a_-_L% RH_ − R_a_-_H% RH_)/R_a_-_L% RH_ (3)

Baseline resistance ratio (%) = R_a_-_H% RH_/R_a_-_L% RH_ (4)

where R_a_-_L% RH_ and R_a_-_H% RH_ respectively present the steady baseline resistance under low-humidity and high-humidity conditions.

Response drift (%) = (S_L% RH_ − S_H% RH_)/S_L% RH_ (5)

Response ratio (%) = S_H% RH_/S_L% RH_ (6)

where S_L% RH_ and S_H% RH_ respectively denote the response value of the target gas under low-humidity and high-humidity conditions. 

In particular, for humidity compensation based on data process, the error rate is used to quantify the accuracy of the calculated concentration of target gas under humid conditions, which is defined as Equation (7):

Error rate = (C_calculate_ − C_real_)/C_real_ (7)

where C_calculate_ and C_real_ respectively denote the calculated and real concentration values of the target gas. 

### 3.1. Surface Engineering

In regard to the gas-sensing centers of sensors, sensing materials directly determine the sensing performance and humidity tolerance [52,53]. Therefore, surface engineering on these materials is the most straightforward method, including noble metals addition, element doping, modification with hydrophobic materials, composites with hydrophilic materials, and post-treatment.

#### 3.1.1. Functionalization of Noble Metals

**Figure 3:**
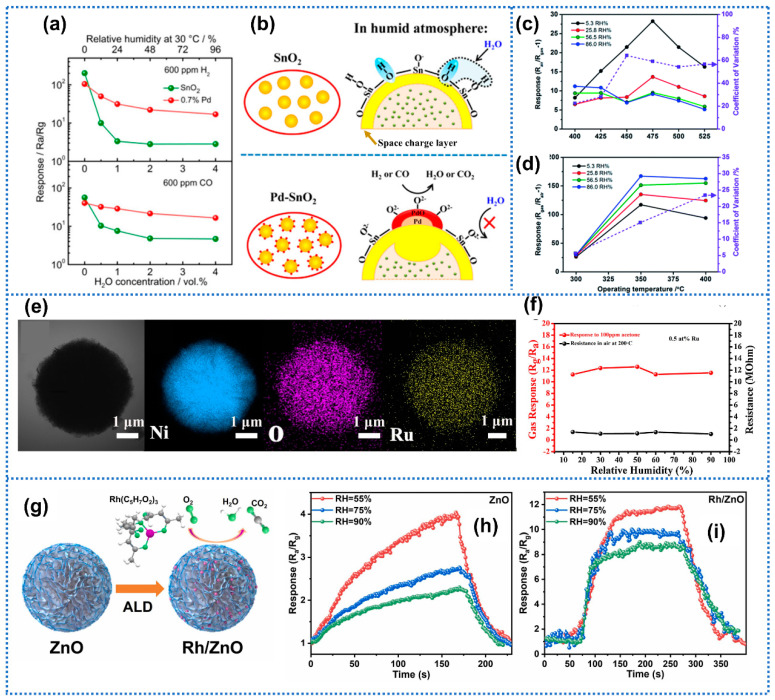
(**a**) Sensor response to 600 ppm H_2_ and CO for SnO_2_ and 0.7% Pd−SnO_2_ in different humidity conditions at 300 °C. (**b**) Schematic diagram of the gas-sensing model for pure SnO_2_ and Pd-SnO_2_ in humid environments. Reprinted with permission from Ref. [58]. Copyright 2015, American Chemical Society. The response and corresponding CV value of 49.4 ppm benzene gas with different RH environments at different operating temperatures of (**c**) pure Sn-ZnO sensor and (**d**) Au-decorated Sn−ZnO sensor. Reprinted with permission from Ref. [60]. Copyright 2017, The Royal Society of Chemistry. (**e**) TEM images and corresponding elemental mapping images of 0.5 at% Ru−doped NiO sample. (**f**) Baseline resistance and gas response to 100 ppm acetone of the 0.5 at% Ru−doped NiO sensor as a function of relative humidity. Reprinted with permission from Ref. [63]. Copyright 2020, Elsevier. (**g**) Schematic synthesis of Rh/ZnO flower-like nanostructures; the response of sensors to 10 ppm TMA under different RH environments, (**h**) Pure ZnO, and (**i**) Rh/ZnO sensors. Reprinted with permission from Ref. [41]. Copyright 2022, Elsevier.

Noble metals (Au, Ag, Pd, Pt, etc.) are widely applied in surface modifications for gas-sensing materials to promote the sensitivity of sensors due to their chemical sensitization and electronic sensitization effect [54,55]. In addition, they are well-known as effective additions for suppressing the poisoning effect of water vapor [56,57]. Moreover, the anti-humidity mechanism of loading noble metal dopants could be summarized into three principles: (I) Promoting the content of surface-adsorbed oxygen ions (for metal oxide sensor); (II) catalytic effects on the gas-sensing reactions; and (III) strong affinity for water molecules. Ma et al. [58] synthesized Pd-functionalized SnO_2_ sensors and investigated the gas-sensing performance toward H_2_ and CO in moist atmospheres. The Pd-SnO_2_ sensor with 0.7 mol% Pd loading exhibited a small response decline toward CO and H_2_ at 300 °C under the humidity range of 0% to 96% RH, significantly surpassing the pure SnO_2_ counterpart, as shown in Figure 3a. The schematic diagram of the gas-sensing model for pure SnO_2_ and Pd-SnO_2_ in moisture conditions was shown in Figure 3b. For pure SnO_2_, the OH^−^ groups competitively adsorbed on the SnO_2_ surface and reduced the available adsorption sites for target gas. Additionally, the relationship of electric resistance on partial pressure at 300 °C showed that the oxygen adsorption species on pure SnO_2_ was changed from O^2−^ to O^−^ when exposed to wet conditions from dry atmospheres. In contrast, the mainly adsorbed oxygen species (O^2−^) on Pd-SnO_2_ was unchanged over humidity. For Pd-doped SnO_2_ sensors, the Pd oxide (PdO) on the Pd surface probably provided initial adsorption sites for O^2−^ species, which then reacted with CO and H_2_ molecules. Moreover, the O^2−^ adsorption on PdO was difficult to be affected by water vapors and may restrain OH^−^ adsorption on the SnO_2_ surface. Furthermore, the effect of Pd size and amount on the anti-humidity performance were investigated [59]. The experimental results exhibited that higher amounts and smaller sizes (<3.5 nm) of Pd favored better humidity-independent performance. Yao et al. [60] proposed Au-Sn co-sensitized ZnO layered nanocrystals for benzene detection. Compared with the Sn-ZnO counterparts, the Au-loaded Sn-ZnO sensors delivered less response deviation with a smaller CV value toward 49.4 ppm benzene within a humidity range of 5.3–86% RH and a wide range of operating temperature, as shown in Figure 3c,d. Thereinto the Au-Sn-ZnO sensor presented a small CV value of 5.7% at 300 °C, indicating the capability of precise monitoring toward benzene within different RH environments. For the Au-loaded Sn-ZnO sensors, Au nanoparticles contributed to the humidity tolerance via increasing surface adsorbed oxygen species and catalytic effects on gas reaction [61,62]. Similarly, Yang et al. [63] synthesized Ru-doped NiO_2_ microspheres by a one-step hydrothermal route for acetone detection (Figure 3e). The 0.5 at% Ru-doped NiO sensor exhibited negligible changes in baseline resistance and response to 100 ppm at 200 °C at a wide humidity range of 15–90% RH (Figure 3f). Additionally, the corresponding response drift was 3%, indicating the insignificant influence in humidity. The excellent humidity independence after Ru loading originated from the increased surface-adsorbed oxygen species and catalytic effects on the gas reaction. Qin et al. [64] utilized Ag nanoparticles to decorate silicon nanowire arrays for high response to trace acetone at high ambient humidity. The DFT calculations revealed that the nano-Ag loaded on the surface of Si nanowires served as wet centers, attracting surrounding water vapor molecules preferentially and thus protecting the reaction sites on Si nanowires for acetone sensing. Byoun et al. [65] decorated single-walled carbon nanotubes (SWCNT) with Pt nanoparticles for NO_2_ detection in ambient environments. The Pt-SWCNT sensor exhibited excellent NO_2_ response at a humidity range of 33% to 76% RH. Theoretical calculations unveiled strong hydrogen bonding interactions between PtO_2_ and water molecules. This result indicated that PtO_2_ served as a water adsorbent to protect the SWCNT-based sensing layer from water interference due to the high affinity for the water molecule of PtO_2_. Similarly, Li et al. [41] performed atomic layer deposition (ALD) of Rh on ZnO flower-like nanostructures for trimethylamine (TMA) detection (Figure 3g). The trimethylamine sensing performances of ZnO with/without Rh loading under different humidity conditions were separately shown in Figure 3h,i. The Rh/ZnO sensor presented significantly improved sensing behavior at a humidity range of 55% to 90% RH compared with pure ZnO. Additionally, the enhanced humidity resistance could be correlated to the loaded Rh nanoparticles, which possessed a strong affinity for water molecules and was readily bound to the hydroxyl and hydrogen groups from water decomposition [66]. This process could restrict the direct reaction between water molecules and oxygen species on the ZnO surface and thus protect the adsorption sites for TMA attachment. Benefiting from the intrinsic chemical sensitization and electronic sensitization effect of noble metals in gas sensing, functionalization of noble metals could achieve the compatibility of good humidity tolerance and high sensitivity.

#### 3.1.2. Element Doping

**Figure 4:**
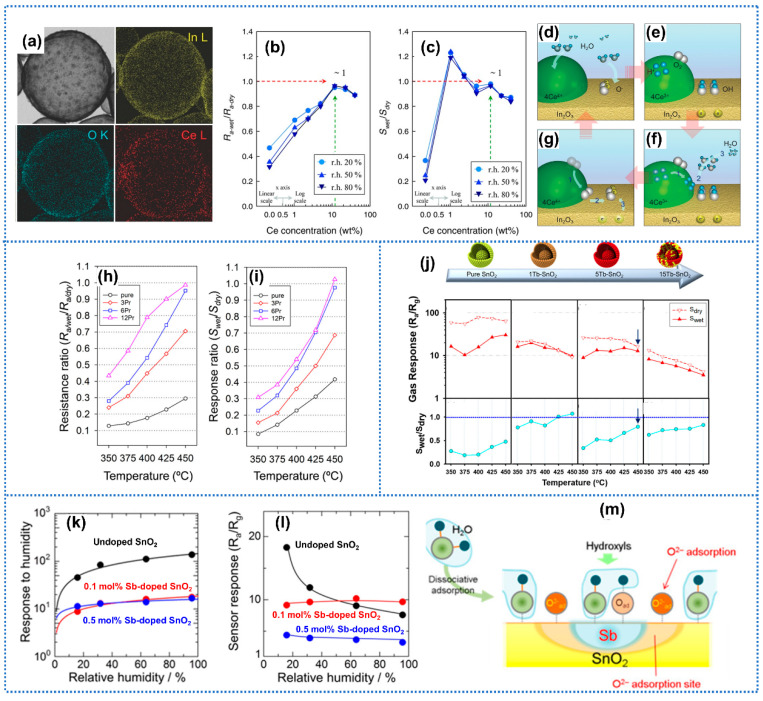
(**a**) Elemental mapping of 11.7 Ce-In_2_O_3_ hollow spheres. (**b**) R_a-wet_/R_a-dry_ and (**c**) S_wet_/S_dry_ of pure and Ce-In_2_O_3_ hollow spheres exposed to 20 ppm acetone at 450 °C. (**d**–**g**) Illustrations of the self-refreshing In_2_O_3_ sensing surface by the CeO_2_ nanoclusters (**d**) water vapor inflow, (**e**) chemisorption, (**f**) desorption, and (**g**) oxygen ion regeneration. Reprinted with permission from Ref. [70]. Copyright 2016, Wiley. (**h**) Resistance ratios (R_a/we_t/R_a/dry_) and (**i**) response ratios (S_wet_/S_dry_) of pure 3Pr−, 6Pr−, and 12Pr−In_2_O_3_ macroporous spheres at 20 ppm acetone measured in the range of 350–450 °C (dry: RH = 0%, wet: RH = 80%). Reprinted with permission from Ref. [71]. Copyright 2019, American Chemical Society. (**j**) Gas responses to 20 ppm acetone of pure SnO_2_, 1Tb−SnO_2_, 5Tb−SnO_2_, and 15Tb−SnO_2_ sensors in the presence of various gases at 350, 375, 400, 425, and 450 °C under dry and 80% RH conditions. Reprinted with permission from Ref. [73]. Copyright 2018, American Chemical Society. (**k**) The humidity dependence of response to humidity at 350 °C and (**l**) humidity dependence of response to 200 ppm H_2_ at 350 °C of undoped SnO_2_, 0.1 mol% Sb-doped SnO_2_, and 0.5 mol% Sb-doped SnO_2_. (**m**) Schematic model of hydroxyl and oxygen coadsorption on Sb-SnO_2_. Reprinted with permission from Ref. [75]. Copyright 2016, American Chemical Society.

Element doping is one important method to promote the sensing performance of gas sensors [67,68]. To enhance the humidity tolerance of sensors, three lanthanide elements praseodymium (Pr), cerium (Ce), and terbium (Tb) with tri/tetravalent ion states (3^+^ and 4^+^) have attracted considerable interest. The coexistence of tri/tetravalent states plays a critical role in alleviating the water-poisoning effect. In detail, due to the valence states, all Pr, Ce, and Tb could proceed reversible oxidation-reduction reactions in humid conditions, which could remove hydroxyl groups from water and facilitate the formation of ionized oxygen species [69]. Yoon et al. [70] synthesized dynamic self-refreshing of In_2_O_3_ sensing surface assisted by Ce doping for humidity-resistant acetone detection. The hollow sphere structure and elemental mapping of 11.7 wt% Ce-In_2_O_3_ (Ce: In = 11.7%) samples were exhibited in Figure 4a. Additionally, the 11.7 wt% Ce-In_2_O_3_ sensor showed negligible baseline resistance drift (R_a-wet_/R_a-dry_ ≈ 1) when humidity changed from dry condition to 80% RH, as shown in Figure 4b. When exposed to 20 ppm of acetone at 450 °C under different humidity conditions, the pure In_2_O_3_ sensor showed a large response drift compared with the dry conditions (63%, 75%, and 79% at RH = 20%, 50%, and 80%, respectively) (Figure 4c). In contrast, the 11.7 wt% Ce-In_2_O_3_ sensor exhibited small response drifts (3% at 20% RH, 4% at 50% RH, and 5% at 80% RH), presenting the remarkable humidity independence. The humidity-tolerant performance of the Ce-doped In_2_O_3_ sensor could be attributed to the regenerative oxidation/reduction reaction of Ce^4+^ and Ce^3+^ as shown in Figure 4d–f. Under moist atmospheres, hydroxyl radicals were generated on In_2_O_3_ when the oxygen species reacted with the H_2_O molecule as shown in Equation (8), and the Ce^4+^ would be reduced to Ce^3+^ and H^+^ via Equation (9). Then, the generated H^+^, OH groups from water decomposition, and Ce^3+^ reacted together using Equation (10).

(8) $$H_{2}O+O_{\mathrm{In}}^{-}=2{\mathrm{OH}}_{\mathrm{In}}+e_{\mathrm{In}}^{-}$$

4Ce^4+^ + 2H_2_O → 4Ce^3+^ + 4H^+^ + O_2_ (9)

OH_In_ + Ce^3+^ + H^+^ → Ce^4+^ +H_2_O (10)

(11) $$O_{2}\;+e_{\mathrm{In}}^{-}\;\to \;O_{\mathrm{In}}^{-}$$

Moreover, the generated oxygen using Equation (9) could diffuse mainly along the surface to the In_2_O_3_ surface and readily reionize by capturing electrons on the In_2_O_3_ surface using Equation (11), resulting in the oxygen readsorption on In_2_O_3_ (Figure 4g). These processes scavenged the hydroxyl radial, regenerated H_2_O molecules, and supplied oxygen species to the In_2_O_3_ surface, leading to the regenerative refresh of the Ce-doped In_2_O_3_ surface. Similarly, Liu et al. [42] proposed highly sensitive and anti-humidity NO_2_ sensors based on Ce-doped SnO_2_ nanomaterials, which were synthesized by a simple hydrothermal route. When the humidity varied from 35% to 50% RH, the response of 1% Ce/SnO_2_ sensors to NO_2_ scarcely moved. Kim et al. [71] synthesized Pr-doped In_2_O_3_ macroporous spheres by ultrasonic spray pyrolysis for acetone detection. Compared with pure In_2_O_3_, the 12 at% Pr-doped In_2_O_3_ sensors presented almost drift-free baseline resistance (R_a/wet_/R_a/dry_ ≈ 1) and unvaried response to acetone at 450 °C (S_wet_/S_dry_ ≈ 1) when humidity varied from 0% to 80% RH, as shown in Figure 4h,i. The humidity-independent performance of Pr-doped In_2_O_3_ could be attributed to the Pr^3+^/Pr^4+^ redox pairs, which promoted the scavenging of surface hydroxyl groups, and regenerative oxygen species adsorption by the following reverse reaction of water poisoning (Equations (12)–(14)):

H_2_O + O^−^ → 2OH + e^−^ (12)

Pr^3+^ + 2OH → Pr^4+^ + H_2_O + O_ad_^−^ (13)

Pr^4+^ + e^−^ → Pr^3+^ (14)

Similarly, Fan et al. [72] doped Co_3_O_4_ with Pr using an electrospray for acetone detection. After Pr doping, the Pr-Co_3_O_4_ sensors showed remarkable humidity independence with unchanged response to 20 ppm acetone at 300 °C at a wide humidity range of 30–90% RH. In contrast, the undoped Co_3_O_4_ sensor presented a significantly larger response drift of 68% under the same conditions. Moreover, Kwak et al. [73] proposed Tb-doped SnO_2_ yolk-shell spheres for acetone sensing. The 5Tb-SnO_2_ (molar ratio of [Tb]/[Sn] was 0.05) sensor surpassed the pure SnO_2_ in humidity tolerance with a lower response drift (20% vs. 52%) value toward 20 ppm acetone at 450 °C when humidity changed from a dry condition to 80% RH (Figure 4j). Similar to Pr and Ce, the regenerative surface refreshing of the SnO_2_ sensors by the reaction between the Tb^3+^/Tb^4+^ redox pairs and surface OH groups from water vapor could account for the humidity-tolerant acetone sensing performance. In addition to the single-lanthanide doping, Kim et al. [74] proposed a Pr-Ce co-doped WO_3_ gas sensor to recognize trimethylamine. Due to the doping of abundant trivalent lanthanides, the Pr-Ce co-doped WO_3_ sensor presented a high response retention nearly reaching 1.0 of S_80% RH_/S_dry_ at 300 °C, significantly surpassing the value (0.14–0.32) of pure WO_3_ counterpart. In addition to lanthanide elements, other metal elements were employed to reduce the water-poisoning effect of gas sensors. Suematsu et al. [75] prepared Sb-doped SnO_2_ gas sensors for H_2_ detection with good humidity independence. The Sb-doped SnO_2_ gas sensor exhibited a significantly smaller response to humidity compared with pure SnO_2_ as shown in Figure 4k. The response value of 0.1 mol% Sb-doped SnO_2_ sensor to 200 ppm H_2_ remained nearly constant at 350 °C independent of the humidity variation (Figure 4l). Within Sb-doped SnO_2_, Sb served as hydroxyl absorbers to preferentially capture the hydroxyl and then generate oxygen species around Sb atoms to some extent as shown in Figure 4m. Therefore, the oxygen species adsorption sites for gas sensing were maintained even in humidity condition. Additionally, aluminum (Al) ions were incorporated into SnO_2_ nanoparticles to obtain humidity-independent ethanol detection [76]. In this scenario, Al ions served as a water vapor absorber on SnO_2_ surface to protect the adsorbed oxygen species under humidity due to the high affinity for water and hydroxyl of Al ions. Although the humidity independence was significantly enhanced by element doping, we noticed in the above studies that the recession of sensitivity was one common phenomenon after element doping. Therefore, if utilizing this method, a trade-off should be made between sensitivity and humidity tolerance.

#### 3.1.3. Modification with Hydrophobic Materials

**Figure 5:**
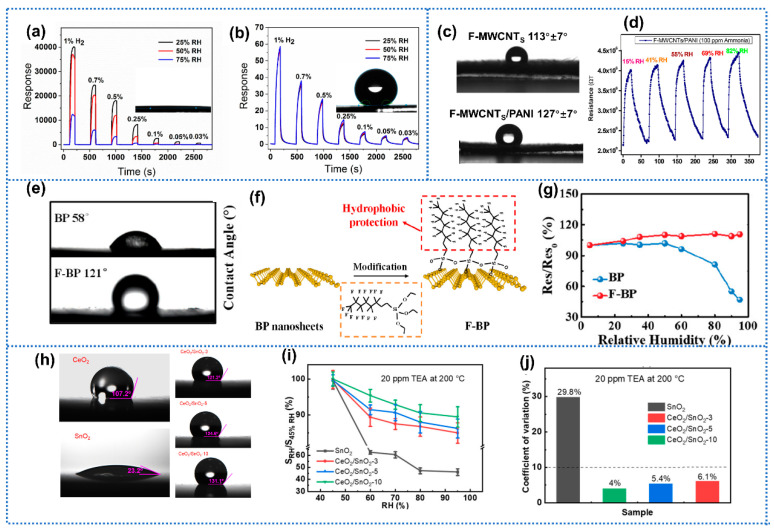
The real-time responses of pristine Pd/TiO_2_ NTs and Pd/TiO_2_ NTs coated by PDMS for 7 h to different concentrations of H_2_ under different RH environments at 25 °C. The insets of (**a**,**b**) showed a water droplet on each surface. Reprinted with permission from Ref. [83]. Copyright 2020, Elsevier. (**c**) Water contact angles of the MWCNTs and MWCNTs/PANI on fabric substrates. (**d**) The dynamic curves of the MWCNTs/PANI sensor at different RH% (15–82%) for 1 ppm NO_2_. Reprinted with permission from Ref. [84]. Copyright 2018, American Chemical Society. (**e**) Water contact angle of BP and F-BP films. (**f**) Schematic illustration of the preparation of F-BP. (**g**) Relative sensing response of the sensor to NO_2_ with different RH environments, where R_es0_ is the response in dry air and R_es_ is the response in humidity. Reprinted with permission from Ref. [89]. Copyright 2021, American Chemical Society. (**h**) Water contact angle of SnO_2_, CeO_2_, and CeO_2_/SnO_2_ films. (**i**) Plots of response retentions for different sensors as a function of RH. (**j**) CV of different sensors under RH in the range of 45−96%. Reprinted with permission from Ref. [100]. Copyright 2022, American Chemical Society.

Many gas-sensing materials suffer from humidity perturbation due to their intrinsic hydrophilicity [77,78]. Therefore, modification with hydrophobic coatings is viewed as one simple strategy to overcome this issue. The surface wettability of materials could be evaluated by the water contact angle [79]. Generally, a contact angle of less than 90° indicates hydrophilic interaction, while an angle greater than 90° indicates a hydrophobic one [80]. In addition, the larger water contact angles indicate stronger hydrophobicity. Frequently, hydrophobic organic materials are coated on materials’ surfaces to enhance the humidity endurance [81,82]. Gao et al. [83] reported PDMS coating Pd-TiO_2_ nanotubes for H_2_ detection with high-humidity immunity and long-term stability. Due to the intrinsic hydrophilic characteristic of TiO_2_, the pristine Pd/TiO_2_ exhibited a superhydrophilic surface with a contact angle of ~0° (Figure 5a, inset). After PDMS coating, the PDMS-Pd/TiO_2_ sample presented a high contact angle of 150° (Figure 5b, inset), indicating significantly enhanced hydrophobicity. Additionally, the PDMS-Pd/TiO_2_ sensor exhibited almost the coincident sensing behaviors at a range of 25–75% RH, superior to PDMS-free sensors, as shown in Figure 5a,b. Over the probed humidity range, the drift in baseline resistance was lower than 20%, which was significantly smaller than the pristine Pd/TiO_2_ sensor (>150%). Qu et al. [25] prepared PDMS-coated CoSnO_3_@MOF for humidity-independent gas detection. The water contact was about 0° of CoSnO_3_@MOF. When coating PDMS, the water contact angle of CoSnO_3_@MOF samples increased to 120°, signifying the conversion of hydrophilicity to hydrophobicity for the sensing film. Maity et al. [84] prepared polyaniline (PANI) functionalized multiwall carbon nanotubes (MWCNTs) by in situ chemical polymerization process of aniline for NH_3_ detection. Moreover, the PANI-coated MWCNTs exhibited a larger water contact angle of 127° on the fabric substrate than pure MWCNTs (113°) as shown in Figure 5c. In terms of NH_3_-sensing performance, the PANI-MWCNTs sensors obtained response values to 100 ppm NH_3_ of 78, 83, 88, 91, and 97 at 15%, 41%, 55%, 69%, and 82% RH, respectively (Figure 5d). Furthermore, the corresponding standard deviation of this response value was 6.5%, indicating excellent humidity resistance. Similarly, Liu et al. [85] proposed PANI-functionalized Bi_2_MoO_6_ sensor for NH_3_ detection. The PANI-Bi_2_MoO_6_ sensor exhibited nearly constant sensing behavior to 1 ppm NH_3_ and negligible fluctuations of baseline resistance when humidity varied from 40% to 90% RH. Liu et al. [86] proposed 3-aminopropyltriethoxysilane (APTES)-functionalized thin-wall porous WO_3_ nanotubes for highly selective NO_2_ detection. The amino groups from APTES selectively reacted with NO_2_ molecules, while the methoxy groups presented highly hydrophobic properties [87,88]. Apart from the increased water contact angle, the APTES-WO_3_ sensor exhibited impervious response to 1 ppm NO_2_ under different RH environments. Xu et al. [89] utilized end-group modification with fluoroalkylsilane on black phosphorus (BP) for enhanced NO_2_-sensing performance. As exhibited in Figure 5e, the pure BP sample possessed a hydrophilic surface, whereas the fluoroalkylsilane functionalized black phosphorus (F-BP) film showed a hydrophobic one due to the water-repelling end groups (-CF_3_ and -CF_2_) of fluoroalkylsilane (Figure 5f). Compared with pure BP, the fluorinated BP sensor presented significantly smaller response drift to 1 ppm NO_2_ (~5% vs. ~55%) over the investigated humidity range (Figure 5g). Qin et al. [90] employed octadecyltrichlorosilane (OTS) functionalized porous Si nanowires to detect trace NO_2_. Thanks to the superhydrophobic structure of OTS, the OTS-Si sensors still showed a discernable signal rather than pure Si ones at 75% RH. Polyvinylidene fluoride (PVDF) was sometimes adopted as hydrophobic coating for humidity-independent NO_2_ detection [91].

In addition to organic materials, inorganic alternatives are useful to promote the humidity tolerance of gas sensors. Therefore, carbon materials were widely used in combination with other materials to construct hydrophobic sensing films [92]. Gu et al. [93] reported one humidity-independent NO_2_ sensor based on In_2_O_3_ nanoflowers decorated with graphite nanoflakes. Owing to the intrinsic hydrophobicity of graphite, the adsorption of water molecules on pure In_2_O_3_ could be effectively blocked. Shboul et al. [94] proposed one anti-humidity gas sensor based on In_2_O_3_ with loading of both inorganic and organic decorated hydrophobic materials, namely, graphite flakes (Gt) and polystyrene (PS). The nanocomposites sensor (In_2_O_3_-10 wt% Gt-17 wt% PS) showcased a negligible response decline in humidity when humidity increased from 25% to 86% RH, suggesting a good humidity tolerance. Singh et al. [95] synthesized WS_2_/MWCNT composites for humidity-tolerant discrimination of NH_3_ owing to the hydrophobic feature of MWCNT. Wang et al. [96] prepared carbon (C) modified coral-like WO_3_ for H_2_S detection. Due to the hydrophobic carbon layers, the WO_3_-C sensors exhibited a small response drift of 10% toward H_2_S when humidity changed from 20% to 98% RH. Huang et al. [97] synthesized one highly sensitive gas-sensing ink based on sulfonated rGO (S-rGO) decorated with SnS_2_ nanosheets for NO_2_ monitoring. For the pure S-rGO sensor, the response to 1 ppm NO_2_ sharply declined with a response drift value of 80% when humidity changed from 30% to 90% RH. In contrast, no clear degradation of sensitivity to NO_2_ appeared in the SnS_2_-decorated S-rGO sensor. The enhanced humidity independence could be attributed to the hydrophobic SnS_2_, which restricted the contact between water and graphene. The hydrophobicity of SnS_2_ came from the outer S atoms, which were reluctant to form hydrogen bonds with water. Lou et al. [98] synthesized ZrO_2_-decorated SnO_2_ porous film for selective trimethylamine (TEA) detection. The three-dimensionally ordered microstructure (3DOM) ZrO_2_-SnO_2_ sample exhibited a larger water contact angle of 60–72° than pure SnO_2_ (26–29°), indicating the enhanced hydrophobicity. This was due to the fact that the continuous 3DOM ZrO_2_ layers could serve as an air hydrophobic layer to restrict water adsorption. Kim et al. [99] proposed Y_2_O_3_-decorated SnO_2_ nanofibers for trace NO_2_ detection. After introducing Y_2_O_3_, the water contact angle of the sensing film increased while the humidity effect on sensor performance was remarkably suppressed. Zhu et al. [100] utilized hydrophobic CeO_2_ to decorate SnO_2_ film for trimethylamine (TEA) detection. As shown in Figure 5h, after introducing CeO_2_, the CeO_2_-SnO_2_ nanocomposites exhibited remarkable enhancement of hydrophobicity compared with the pure SnO_2_ sample. In contrast to the significant response decline in pure SnO_2_ sensor toward TEA under moisture conditions (Figure 5i,j), the CeO_2_-decorated SnO_2_ sensors achieved nearly 90% response retention and a small coefficient of variation (~5%). Of note, among these hydrophobic materials utilized in the above reports, some hydrophobic materials could selectively react with target molecules (such as APTES and PANI) or form heterojunctions with gas-sensing materials (such as ZrO_2_-SnO_2_ and CeO_2_-SnO_2_). Modification with these hydrophobic materials is one popular method to promote the sensitivity of sensors. Therefore, the balance between high sensitivity and excellent humidity resistance could be realized by utilizing proper hydrophobic materials.

#### 3.1.4. Modification with Hydrophilic Materials

**Figure 6:**
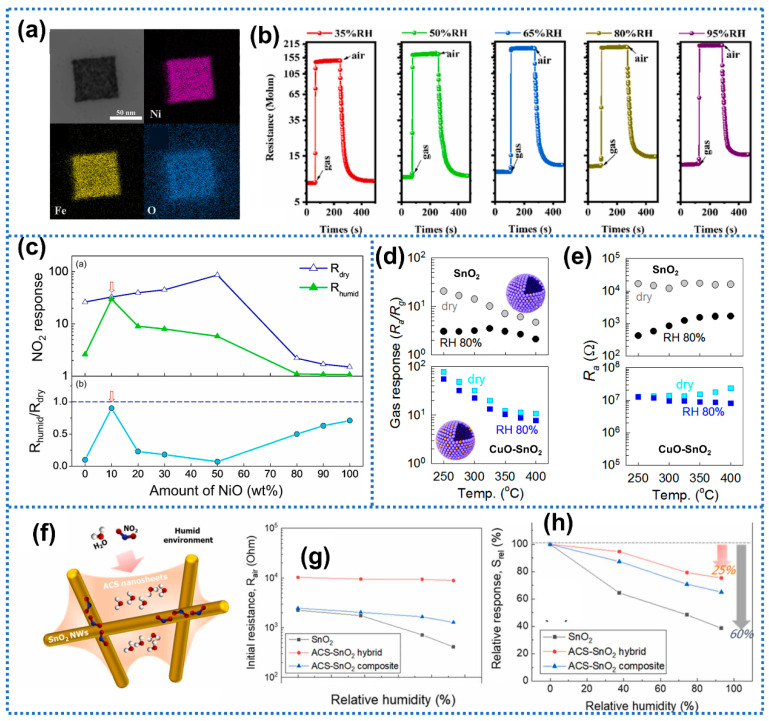
(**a**) The elemental mapping images of NiO/NiFe_2_O_4_-1.5. (**b**) Dynamic sensing curves of NiO/NiFe_2_O_4_-1.5 sensor toward 100 ppm acetone at the working temperature of 200 °C under different RH environments. Reprinted with permission from Ref. [102]. Copyright 2022, Elsevier. (**c**) Responses of NiO-ZnO composites depending on NiO contents under dry (open triangle) and humid (filled triangle) atmosphere and the R_humid_/R_dry_ values of the composite NiO-ZnO composites depending on NiO contents. Reprinted with permission from Ref. [103]. Copyright 2021, Elsevier. Gas-sensing characteristics of pure and 2.0 at% CuO-loaded SnO_2_ hollow spheres. (**d**) Gas response (R_a_/R_g_) to 1 ppm H_2_S and (**e**) baseline resistance in air (R_a_). Reprinted with permission from Ref. [104]. Copyright 2014, Elsevier. (**f**) Schematic illustration showing the water-trapping effect of 2D ACS nanosheet in the spider-web-like SnO_2_ NWs. (**g**) Baseline resistance and (**h**) relative response (S_rel_ = S_wet_/S_dry_) of SnO_2_ NWs, ACS-SnO_2_ hybrid, and ACS-SnO_2_ composite at 150 °C to 10 ppm NO_2_. Reprinted with permission from Ref. [43]. Copyright 2022, Elsevier.

Of note, the presence of water vapor in atmospheric air is inevitable; therefore, introducing absorbents to capture water molecules is one simple strategy. Hydrophilic additives could serve as an effective water molecule reservoir to protect gas-sensing centers from humidity interference. Kim et al. [101] proposed NiO-doped hierarchical SnO_2_ for CO detection. After doping from 0.64 to 1.27 wt% NiO, the NiO-SnO_2_ sensors exhibited negligible humidity dependence toward 50 ppm CO at 375 °C when sensors were exposed to dry and 25% RH atmosphere, respectively. The diffuse-reflectance Fourier transform infrared measurements revealed that most of the water-driven species were predominantly absorbed by NiO rather than SnO_2_. Therefore, the interaction between water molecules and the SnO_2_ surface was effectively blocked within NiO-SnO_2_ sensors, allowing for sufficient oxygen species on SnO_2_ to react with CO even in humid conditions. Taking advantage of the good affinity of NiO to water, Yang et al. [102] synthesized metal-organic frameworks (MOFs) derived porous NiO/NiFe_2_O_4_ nanocubes for acetone detection (Figure 6a). The NiO/NiFe_2_O_4_ sensor presented similar dynamic resistance curves toward 100 ppm acetone in a wide humidity range of 35–95% RH as shown in Figure 6b. The response drift (S_35% RH_−S_95% RH_)/S_35% RH_) of NiO/NiFe_2_O_4_ sensor was only 9.5%, indicating the negligible humidity interference. Furthermore, Jin et al. [103] investigated the effect of NiO content within NiO-ZnO composites on the humidity-tolerance during gas detection. As shown in Figure 6c, the 0.1 NiO-0.9 ZnO sensor (the weight ratios of the two precursor materials ([Ni]:[Zn] = 10:90) presented a nearly similar response to NO_2_ under dry and 81% RH conditions. With further increase in NiO content, the response of NiO-ZnO sensor toward NO_2_ exhibited a clear response decrease at 81% RH. Therefore, excessive content of hydrophilic NiO would weaken the humidity independence of NiO-ZnO sensors. As a member of transition metal oxides, CuO possesses similar physic-chemical properties to NiO, especially the high affinity toward water. Choi et al. [104] synthesized CuO-loaded SnO_2_ hollow spheres by ultrasonic spray pyrolysis for H_2_S detection in highly humid atmosphere. The 2.0 at% CuO-loaded SnO_2_ sensor exhibited favorable humidity-resistant features than pure SnO_2_, as shown in Figure 6d,e. Additionally, the CuO-SnO_2_ sensor exhibited clear signals toward 0.2–1 ppm H_2_S at 80% RH, indicating the ability to diagnose halitosis (0.1–0.5 ppm H_2_S) via human breath. Similarly, Sun et al. [105] synthesized clustered CuO/In_2_O_3_ nanospheres with different CuO contents as sensing materials for CO detection, and found that the CuO introduction could alleviate the humidity effect. Cho et al. [43] introduced two-dimensional calcium silicate (CS) nanosheets to decorate SnO_2_ nanowires for NO_2_ detection. The CS nanosheets could adsorb water molecules on their surface through the hydrogen bond and thus be used as one water adsorbent (Figure 6f). As exhibited in Figure 6g, the anhydrous CS-decorated SnO_2_ (ACS-SnO_2_) sensors rather than pure SnO_2_ displayed the same base resistance regardless of humidity variation. In regard to the NO_2_ sensing performance, the ACS-SnO_2_ sensor showed a response drift of 25% at 93.1% RH compared with dry conditions, which was significantly smaller than pristine SnO_2_ sensor (66%) (Figure 6h). In addition, SiO_2_ is widely utilized to improve the humidity tolerance of gas sensor due to its hydrophilic feature [106]. Of note, the content of hydrophilic materials should be optimized in composites; otherwise, excessive hydrophilic materials would be introduced over a large number of water molecules into the sensing films and thus lead to a recession of humidity tolerance.

#### 3.1.5. Post-Treatment

**Figure 7:**
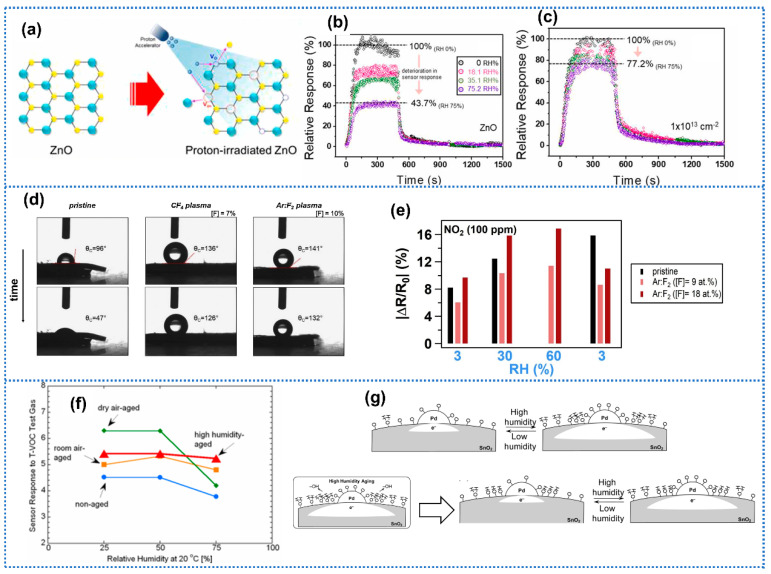
(**a**) Schematic diagram of proton-irradiated ZnO gas sensors. Dynamic relative response plots of different gas sensors to 10 ppm NO_2_ at 300 °C under various humidity conditions. (**b**) Pristine ZnO and (**c**) 1 × 10^13^ protons/cm^2^ proton-irradiated ZnO. Reprinted with permission from Ref. [107]. Copyright 2021, Elsevier. (**d**) Water contact angle of pristine carbon nanotubes and fluorinated carbon nanotubes in 30 s. (**e**) The relative sensor response to 100 ppm NO_2_ at variable humidity conditions for pristine and Ar:F_2_ plasma fluorinated carbon nanotubes with different fluorination yields. Reprinted with permission from Ref. [108]. Copyright 2019, Elsevier. (**f**) Non, dry air, room air, and high-humidity aged Pd/SnO_2_ under different humidity conditions and 400 g/m^3^ T–VOC test gas concentration. (**g**) Schematic images of the surface conditions of Pd-loading area on the non-aged Pd/SnO_2_ and high-humidity aged Pd/SnO_2_. Reprinted with permission from Ref. [111]. Copyright 2010, MDPI.

Appropriate post-treatment is another viable strategy to improve the humidity tolerance of sensing materials. Bang et al. [107] performed proton-beam engineered surface-point defects on ZnO for NO_2_ detection under humid conditions as shown in Figure 7a. When sensors were exposed to 10 ppm NO_2_ at 300 °C with humidity changing from 0% to 75% RH, the ZnO irradiated with the fluency of 1 × 10^14^ protons/cm^2^ sensor exhibited a response drift of 23.8%, significantly smaller than pure ZnO of 56.3%, presenting an enhanced humidity resistance (Figure 7a,c). The XPS and PL results found that three types of vacancy defects (V_O_, V_Zn_, and V_Zn-O_) were induced after the proton irradiation. The DFT calculations found that the defect-free ZnO surface possessed higher adsorption energy with H_2_O than NO_2_ (−1.146 eV vs. −0.527 eV), indicating the preferential adsorption of H_2_O on the ZnO surface. In contrast, all these vacancy defects presented higher adsorption energy with NO_2_ than H_2_O molecules, revealing that NO_2_ molecules were preferentially adsorbed on the defective ZnO surfaces compared with H_2_O. Struzzi et al. [108] performed a controlled plasma fluorination for carbon nanotubes to form a superhydrophobic surface using two different fluorine-based gas precursors (Ar:F_2_ and CF_4_). For pure carbon nanotubes, the water contact angle changed from 96° to 47° in 30 s. In contrast, the water contact angles of fluorinated carbon nanotubes remain at more than 125° in 30 s, indicating the promoted hydrophobic property by functionalization. In regard to the gas-sensing performance, no response to 100 ppm NO_2_ could be observed for pure carbon nanotubes at high humidity of 60% RH. Whereas, the fluorinated carbon nanotubes could remain at a high response to NO_2_ at a wide humidity range from 3% to 60% RH. Du et al. [109] adopted fluorocarbon plasma treatment on In_2_O_3_ for NO_2_ detection. The fluorocarbon-modified In_2_O_3_ exhibited a hydrophobic surface with a water contact angle of 137°, which was significantly higher than pure In_2_O_3_ (16°) due to the hydrophobic character of fluorocarbon. When sensors were exposed to 1 ppm NO_2_ with humidity changing from 47% to 92% RH, the fluorocarbon-modified In_2_O_3_ showed a small coefficient of variation (CV) of 3.7% for the response, significantly smaller than pure In_2_O_3_ (60.5%), presenting a significant improvement in humidity independence. Li et al. [110] performed chemical etching on SnO_2_/Zn_2_SnO_4_ to enhance the humidity resistance in H_2_O_2_ detection. The Fourier transform infrared spectroscopy (FTIR) of etched SnO_2_/Zn_2_SnO_4_ samples showed significantly smaller adsorption peak signals than pure SnO_2_/Zn_2_SnO_4_ in the range of 3000–3600 cm^−1^ and 1500–1600 cm^−1^, which separately originated from the OH stretching and bending vibration peaks of water molecules, indicating enhanced resistance to humidity. Itoh et al. [111] investigated the effect of high-humidity aging on the humidity tolerance of the Pd-doped SnO_2_ sensor. Briefly, the high-humidity aging of the Pd-doped SnO_2_ film was performed in pure air with 90% RH heat-treated at 400 °C for 2 weeks. Compared with the non-aged Pd-doped SnO_2_ sensor, the high-humidity aged sensor exhibited less response fluctuation on exposure to toluene with humidity changing from 25% to 75% RH as shown in Figure 7f. During the high-humidity aging process, the water adsorbed on Pd-doped SnO_2_ surface would bind to the SnO_2_ framework as hydroxyl groups and the water-adsorbable sites were blocked (Figure 7g). Therefore, the amount of water adsorbed was unvaried under different humidity conditions, along with a stable response to VOCs. However, they did not investigate the stability of this method. Further performance of the high-humidity aging on the humidity tolerance should be studied in the future.

### 3.2. Physical Isolation

**Figure 8:**
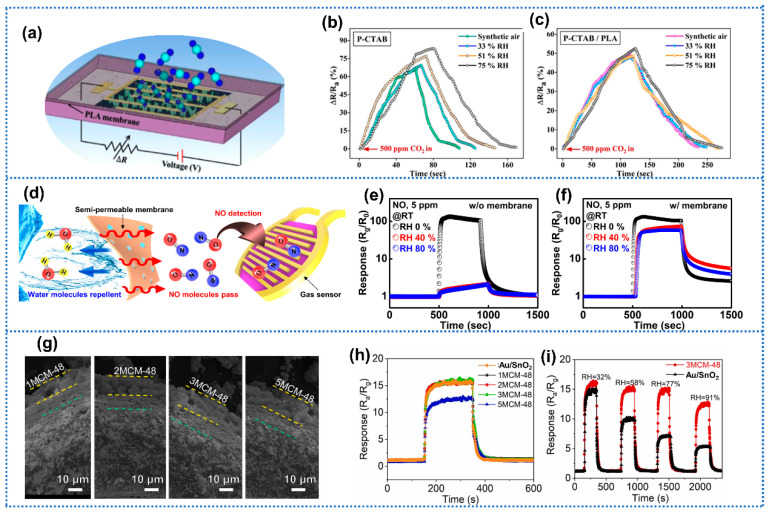
(**a**) Schematic diagram of sensor with PLA membrane coating. Effect of humidity on (**b**) P-CTAB and (**c**) P-CTAB/PLA sensors toward CO_2_. Reprinted with permission from Ref. [112]. Copyright 2020, American Chemical Society. (**d**) Schematic illustration of the principle of enabling gas measurements in humid conditions. Semipermeable membranes (polydimethylsiloxane, PDMS, thickness ~50 μm) selectively allow for the passage of gas molecules while acting as a barrier to water molecules. The dynamic responses of integrated gas sensors (**e**) without and (**f**) with a semipermeable membrane to 5 ppm NO_2_ at several levels of relative humidity. Reprinted with permission from Ref. [44]. Copyright 2020, Springer. (**g**) SEM image of a formaldehyde sensor device with different thickness of MCM-48 layer. (**h**) Response values of the pure Au/SnO_2_, 1MCM-48-Au/SnO_2_, 2MCM-48-Au/SnO_2_, 3MCM-48-Au/SnO_2_, and 5MCM-48-Au/SnO_2_ at 25% RH, respectively. (**i**) Dynamic curve of 3MCM-48-Au/SnO_2_ and Au/SnO_2_ sensors at different RH environments. Reprinted with permission from Ref. [116]. Copyright 2022, Elsevier.

In addition to surface engineering, leveraging waterproof and breathable membranes to insulate moisture from gas-sensing films has been shown to be positive for enhanced humidity resistance. Rathi et al. [112] proposed one polylactic acid (PLA) membrane-protected cetyltrimethylammonium bromide (CTAB)-functionalized black phosphorus (P) for CO_2_ detection. PLA membrane has been widely utilized as a barrier layer of sensors to block water vapor and pollution due to its hydrophilic property [113]. Moreover, the PLA membrane with high crystallinity presented high diffusivity and permeability toward CO_2_ gas [114]. The P-CTAB sensor with PLA membrane (thickness of 0.25 μm) exhibited negligible humidity effect on CO_2_ sensing (Figure 8b,c). Within 1 month, the response to 500 ppm CO_2_ of P-CTAB/PLA sensors remained nearly constant, indicating the remarkable stability. Furthermore, the authors investigated the impact of the thickness of PLA membrane on gas-sensing performances. As the thickness of PLA membrane increased from 0.25 to 0.75 μm, the response to CO_2_ decreased due to the decline in permeability within the thicker PLA membrane. Ko et al. [44] reported a silicon film covered with polydimethylsiloxane (PDMS) membrane for NO_X_ detection. Therefore, the PDMS layer served as a semipermeable membrane to suppress the humidity effect. The methyl groups (Si-CH_3_) on the surface of PDMS decreased the surface energy, and siloxane backbones (Si-O) inside the polymer network formed a pathway for NO diffusion, leading to its hydrophobicity and gas permeability, respectively (Figure 8d). The PDMS-covered sensor exhibited a slight response decline toward 5 ppm NO when humidity changed from 0% to 80% RH, while almost no responses could be observed in the absence of membrane, as shown in Figure 8e,f. This result verified that the PDMS membrane could effectively insulate water vapor from the sensing film. Additionally, Yang et al. [115] adopted a semipermeable PDMS membrane to decorate laser-induced graphene (LIG) for NOx detection. After PDMS membrane coating, the water contact angle of the sensing film changed from 0° to 130°, indicating the transformation from a hydrophilic surface to hydrophobic one. Moreover, NO-sensing performance of PDMS-coated LIG sensor was maintained in the humidity range of 15–90% RH, outperforming the LIG sensor without membrane. Liu et al. [116] prepared a humidity-tolerant formaldehyde gas sensor via layering a molecular sieve MCM-48 on SnO_2_-Au composites (Figure 8g). Here, MCM-48 is one typical mesoporous silica molecular sieve with a three-dimensional spiral channel network structure, allowing for the gas molecules to diffuse through its pores. Furthermore, the siloxane bands in MCM-48 could react with water molecules to form silica-hydroxyl bonds with reversible recovery at a high temperature (>110 °C), which was widely used to capture water vapor from moist air. The thickness of MCM-48 was adjusted by the number of times of the dip-coating process. As shown in Figure 8h, the sensor with less than three times of MCM-48 dip-coating exhibited similar response performance toward 5 ppm formaldehyde at 25% RH, while a clear decline appeared with coating of more than five times. When humidity changed from 32% to 91% RH, the 3MCM-SnO_2_-Au (three times of MCM-48 coating) sensors maintained more than 80% of its initial response value, significantly larger than SnO2-Au without coating (28%), indicating that the 3MCM could effectively reduce the interference of moisture (Figure 8i). Analogously, SBA-15 molecular sieve layer was introduced to the decorated Pt-In_2_O_3_ sensing film for acetone detection [117]. Similar to MCM-48, SBA-15 molecular sieve not only could ensure the rapid diffusion of acetone in its mesoporous structure (pore size of 9 nm), but also could serve as an effective desiccant due to the hydrolysis of siloxane bonds at a wet condition. After coating SBA-15, the response of sensors toward acetone changed only slightly under a wide humidity range (25–100% RH). Zhou et al. [118] covered a commercial waterproof and gas-permeable PTFE membrane on the N-MXene layer to reduce the interference of humidity in NH_3_ detection. Additionally, the sensor response fluctuated slightly over the varying humidity from 13% to 79% RH after membrane incorporation, verifying the feasibility of this strategy to enhance the humidity tolerance. Sayegh et al. [45] covered one porous alumina nanomembrane on SnO_2_ nanowires for humidity-independent NO_2_ detection. The conformal alumina (Al_2_O_3_) thin film was deposited by molecular layer deposition (MLD) on the SnO_2_ nanowires using trimethylaluminum and ethylene glycol as precursors, followed by annealing in air at 400 °C. The thickness of the alumina membrane could be modulated by the cycles of MLD. With the thickness of Al_2_O_3_ increasing from 2 to 10 nm, the Al_2_O_3_-coated SnO_2_ nanowires exhibited increasing humidity tolerance toward 100 ppm NO_2_ when humidity changed from 0% to 90% RH. Moreover, the SnO_2_ nanowires with 10-nm-thick alumina layers exhibited almost the same response value, indicating that the Al_2_O_3_ membrane could block the contact between water molecules and SnO_2_. Similarly, Kondalkar et al. [119] coated the Pt-ZnO nanorod MEMS sensor with a thin nanoscale moisture-blocking conformal Al_2_O_3_ by atomic layer deposition (ALD) for acetylene detection. Furthermore, the Al_2_O_3_-coated Pt-ZnO sensors exhibited almost constant sensing performance toward 200 ppm acetylene with a response drift of 5% in the humidity range from the dry condition to 50% RH, which is significantly better than (36%) of pure Pt-ZnO sensor. Whereas, the diffusion of target gases would be hindered by these membranes or molecular sieves to some extent, and thus lead to a decline in sensitivity. Therefore, the thickness of membranes or molecular sieves should be optimized to achieve humidity-independent performances of sensors with the least loss of sensitivity.

### 3.3. Working Parameter Modulation

**Figure 9:**
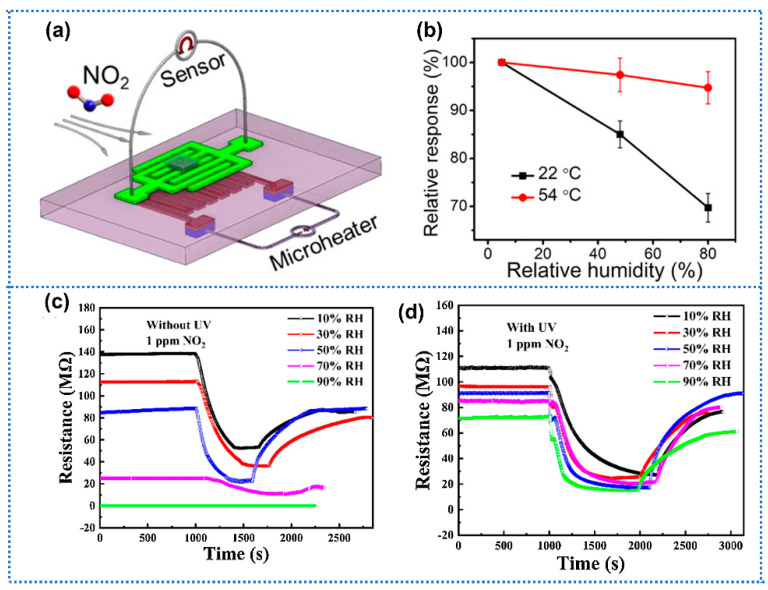
(**a**) Schematic illustration of the synthesis of 3D SnO_2_/RGOH sensor with a microheater. (**b**) Plot of relative responses of SnO_2_/RGOH to 3 ppm NO_2_ vs. RH at 22 and 54 °C, respectively. Reprinted with permission from Ref. [46]. Copyright 2020, American Chemical Society. Sensing characteristics of WS_2_/PbS sensor to 1 ppm NO_2_ at different humidity conditions (**c**) without and (**d**) with UV−light illumination. Reprinted with permission from Ref. [47]. Copyright 2021, Elsevier.

Operating temperature is one important parameter to affect the operation performance of gas sensors in terms of sensitivity, response/recovery time, and selectivity. Additionally, the water adsorption on the sensing film is related to the operating temperature. Wu et al. [46] performed the regulation of operating temperature on NO_2_ sensor based on SnO_2_-graphene hydrogel to improve its humidity tolerance (Figure 9a). When the operating temperature was increased from 20 to 54 °C, the response drifts toward 3 ppm NO_2_ caused by RH changing from 5% to 80% RH reduced from 30.8% to 9.9% (Figure 9b). The elevated operating temperature could facilitate the desorption of water and thus reduce the humidity interference on gas detection. Moreover, the authors improved the humidity immunity of Au-modified graphene hydrogel based NO_2_ sensor by increasing the operating temperature [120]. However, the response to NO_2_ appeared to decline with the elevating operating temperature in their works. In regard to CGS, sensitivity is closely related to the operating temperature. This phenomenon suggested that if the operating temperature does not match the optimal one, elevating the operating temperature for humidity tolerance probably led to sensitivity reduction. Moreover, adjuvant UV light illumination was another feasible method to improve humidity tolerance. Chen et al. [47] introduced UV-light illumination on one NO_2_ gas sensor based on WS_2_/PbS. After introducing UV light, the sensor exhibited less water-susceptible response to 1 ppm NO_2_ over a humidity range of 10–90% RH (Figure 9c,d). Generally, the adsorbed water on the sensing film was not easily removed due to the low thermal energy at room temperature. Whereas, the UV-light illumination would provide more activation energy to promote the desorption of water molecules, and thus improve the humidity immunity of gas sensors.

Furthermore, we summarized the anti-humidity performance of the stated gas sensors via surface engineering, physical isolation, and working parameter modulation in Table 1.

Although these strategies could effectively improve the humidity tolerance, the degradation of response caused by these methods was one common phenomenon, which affected the precise detection of target gases with low concentrations. Therefore, the trade-off between gas sensitivity and humidity independence should be considered before the design of gas sensors. Meanwhile, we found that the perfect compatibility was achieved between anti-humidity and sensitivity within some gas sensors in Table 1. Additionally, the corresponding strategies should be preferentially taken into consideration. 

### 3.4. Algorism Compensation

**Figure 10:**
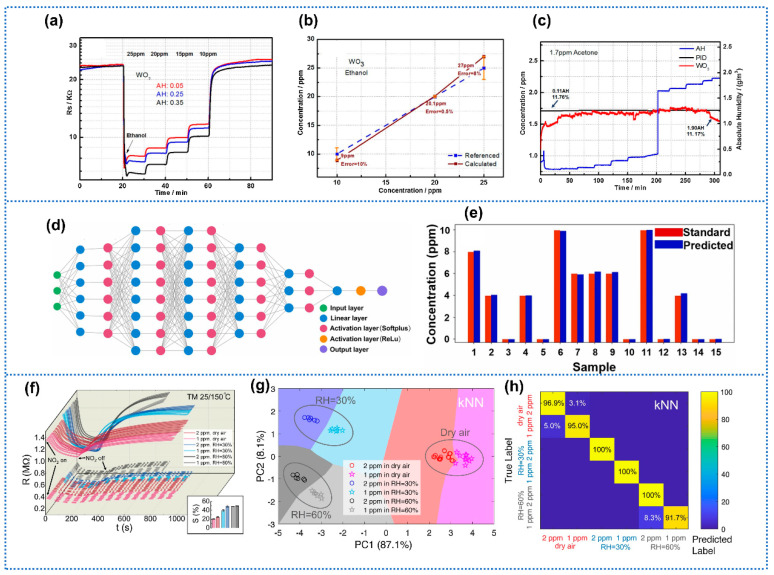
**(a**) Transient resistance of WO_3_ sensor to ethanol with different concentrations and humidity conditions. (**b**) Calculation of concentration of WO_3_ sensor in response to ethanol. (**c**) Comparison of PID sensor and compensation model for WO_3_ sensor. Reprinted with permission from Ref. [121]. Copyright 2021, Elsevier. (**d**) Schematic illustration of deep BP neural network model structure with 14 layers. (**e**) The predicted outputs based on Adam optimizer with Softplus activation function. Reprinted with permission from Ref. [122]. Copyright 2022, Elsevier. (**f**) Repeated measurements of sensor responses of six sets of ([NO_2_], [RH]) paired groups. The inset shows the responsivity of each group. (**g**) PCA scores and classification with kNN. (**h**) Confusion matrix of the classification system. Reprinted with permission from Ref. [49]. Copyright 2020, Elsevier.

In regard to CGS, the interference caused by water vapor not only includes the variation of baseline resistance and response to target gas, but also the response/recovery time. Humidity compensation based on mathematical models and neural network could utilize these features to realize the accurate calculation for target gas concentration. Yan et al. [121] proposed one humidity compensation model based on the power-law response for MOS to detect the vapor of volatile organic compounds. Figure 10a exhibited the dynamic resistance curves of the WO_3_ sensor toward ethanol with different concentrations under different humidity conditions. In brief, the compensation model can be expressed as:

C_gas_ = f (R, AH, T) (15)

where C_gas_ is the concentration of target gas, R denotes the steady resistance in target gas under humid conditions, AH is the absolute humidity value, and T is the operating temperature. When the operating temperature remained unchanged, the model could be simplified as C_gas_ = f (R, AH), indicating that the concentration of target gas could be obtained when the value of R and AH in the model were inputted. It was assumed that the water adsorption on the MOS surface was similar to the oxygen adsorption model, following the power law of response. Additionally, the prediction error of concentration was low in the range of 0.5–10%, indicating the feasibility of this compensation model (Figure 10b). However, compared with the photo ionization detector (PID), the WO_3_ sensor exhibited a larger calculation error at low humidity (<0.11 AH) and high humidity (>1.9 AH) as shown in Figure 10c, reflecting the limit of the compensation model. Wang et al. [122] proposed the self-adaptive temperature and humidity compensation based on deep back propagation neural network for NO_2_ detection (Figure 10d). The steady resistance of 240 samples toward 2–10 ppm NO_2_ (2 ppm as a step) was collected with humidity changing from 20% to 90% RH (10% RH as a step) and temperature changing from 10–50 °C (10 °C as a step). Then, 93.75% of samples were randomly selected as the training set, and the rest of the samples were automatically selected as the development set. Stochastic gradient descent (SGD) algorithm with a mini-batch algorithm was adopted to well balance the model performance and the training time complexity, resulting in 76.68% performance improvement and nearly six times training time reduction after 1000 epochs, respectively. Softplus activation function was combined with Adam optimizer to further improve the model performance with a good recognition accuracy (1.37% relative error, corresponding to 0.0087 mean square error (MSE)) (Figure 10e). Wu et al. [49] proposed a framework which utilized temperature modulation (TM) algorithms and machine learning (ML) approaches employing principal component analysis (PCA) and cluster analysis of transient features to detect NO_2_ concentrations under specific RH conditions. Generally, the dynamic responses comprised various characteristics resulting from gas adsorption/desorption processes, diffusion speeds, and chemical reactions. Additionally, 13 features for dynamic response curves toward different concentrations of NO_2_ at different humidity conditions were extracted. Then, PCA was performed to reduce the dimensionality of the computed features and visualize the clusters formed in low-dimensional projections. The input dataset comprised six sets of ([NO_2_], [RH]) paired groups, in combinations of [NO_2_] = 1, 2 ppm in [RH] = 0%, 30%, 60% RH. The input training set was exhibited in Figure 10f, containing 10–20 repeated tests obtained for three prepared α-Fe_2_O_3_/rGO sensors simultaneously for each group. Figure 10g exhibited the visual representation after PCA transformation. With the two most important principal components, three widely distinct RH classes were separated and each was composed of two distinct NO_2_ clusters, with 95.2% of variances explained. Moreover, the classification system realized the quantification of six classes of ([NO_2_], [RH]) conditions with five classes above 95% accuracy and an overall classification accuracy of 97.3%, as shown in Figure 10h. Oh et al. [48] proposed machine learning-based discrimination of indoor pollutants using In_2_O_3_ gas sensor array with high-humidity endurance. The sensor array (five sensors) was evaluated using PCA and neural network-based classification in terms of the gas sensor data type/amount, neural network algorithms, sensor combinations, and environmental factors. Furthermore, the discrimination of five types of VOCs under different humidity conditions (0%, 30%, 50%, 80% RH) was realized by deep learning algorithms. The above-mentioned algorism compensations were based on a large amount of data in regard to the features of sensors. Therefore, sensors with high stability are very important to ensure constant sensing features for a long time.

### 3.5. Novel Material Development

**Figure 11:**
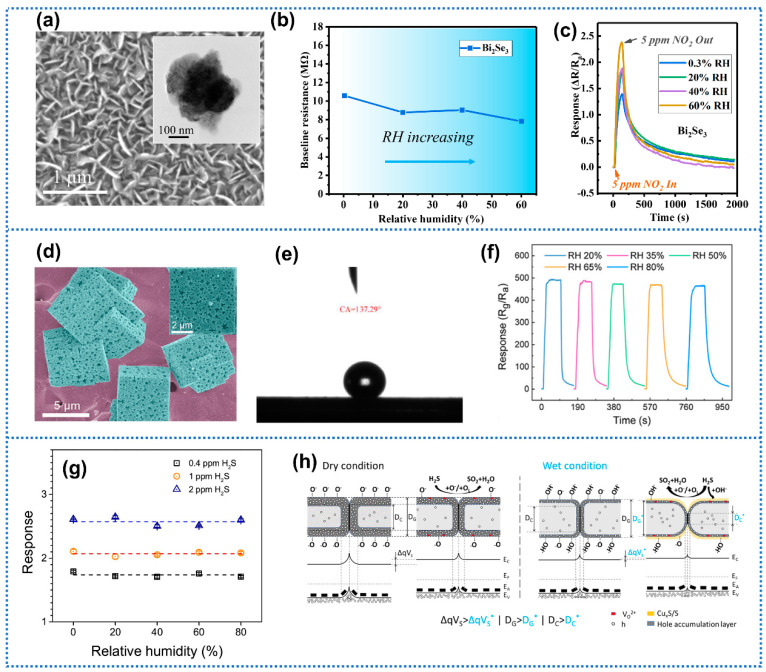
(**a**) SEM image of free−standing Bi_2_Se_3_ nanoplates, inset: TEM image of single Bi_2_Se_3_ nanoplate. (**b**) Baseline resistance of Bi_2_Se_3_ sensors at different humidity conditions. (**c**) Dynamic gas-sensing transients of the Bi_2_Se_3_ sensor to 5 ppm NO_2_ at different humidity conditions. Reprinted with permission from Ref. [50]. Copyright 2022, Elsevier. (**d**) SEM image of the porous In_2_O_3_ sample. (**e**) Water contact angle of the porous In_2_O_3_ sample. (**f**) Dynamic gas-sensing transients of the In_2_O_3_ sensor to 50 ppm NO_2_ as a function of RH at room temperature. Reprinted with permission from Ref. [51]. Copyright 2021, American Chemical Society. (**g**) Sensor responses toward different H_2_S concentrations as a function of relative humidity. (**h**) Schematic of the CuO nanosheet sensing layer from the aspects of morphology, energy band, and surface reactions under dry and humid conditions. Reprinted with permission from Ref. [130]. Copyright 2020, Elsevier.

As traditional sensing materials could not well meet the demanding scenario requirements of future gas sensors, developing novel nanomaterials or new nanostructures is one promising strategy to improve the humidity tolerance of gas sensors. Yuan et al. [123] synthesized Ag_2_Te nanowires for trace NO_2_ detection. When the Ag_2_Te sensor was exposed to 1 ppm NO_2_ with humidity changing from 50% to 90% RH, the sensor exhibited a small fluctuation of baseline resistance drift of 1.84% and response drift of 14.14%, suggesting a remarkable humidity tolerance. AgTe_2_ did not possess any hydrophilic functional groups, such as oxygen-containing functional groups anchored on the surface, which provided the operation stability in humid conditions. Our group initiatively proposed one humidity tolerant NO_2_ sensor based on nanoplate Bi_2_Se_3_ film at room temperature as shown in Figure 11a [50]. Due to the inherent hydrophobicity, the Bi_2_Se_3_ sensor exhibited excellent humidity resistance at a humidity range of 0.3–60% RH with a small baseline drift of 26.1% (Figure 11b). In contrast to the response decline caused by humidity interference, the response of Bi_2_Se_3_ sensor toward NO_2_ (5 ppm) was enhanced under moist conditions (Figure 11c), which indicated the inspiring NO_2_ detection potential in high-humidity atmospheres. Liu et al. [51] synthesized hydrophobic 3D porous In_2_O_3_ microcubes for NO_2_ recognition. The water contact angle of the porous In_2_O_3_ microcubes samples was 137.29°, showing excellent hydrophobicity (Figure 11d). The hydrophobic surface restricted the interference of humidity in NO_2_ detection even though the humidity changed from 20% to 80% RH as shown in Figure 11e. Wu et al. [124] proposed 3D superhydrophobic reduced graphene oxide (rGO) for NO_2_ sensing with enhanced endurance to humidity. The 3D rGO with unique hierarchical structures was synthesized by the reduction in graphene oxide (GO) through spark plasma sintering (SPS). After the reduction process, oxygenate functional groups in GO were effectively removed to a minimal content of 8.8%, leading to the sharp decrease in the surface energy of GO materials. Therefore, the rough surface with unique hierarchical structures and reduced surface energy of the prepared rGO sample led to the transformation from hydrophilic GO to superhydrophobic rGO (water contact angle: 53° vs. 154°). Moreover, the response of rGO sensors to 1 ppm NO_2_ exhibited a small decline of 5% when the humidity changed from 0% to 70% RH, indicating excellent resistance to humidity. Furthermore, Li et al. [125] utilized the hydrophobic advantage of rGO to prepare a triethylamine gas sensor with high-humidity tolerance. Over the last decades, *p*-type metal oxide-based gas sensors, such as CuO, NiO, and Co_3_O_4_ have received significantly less attention compared with *n*-type metal oxides due to their lower sensitivity [126]. Inspiringly, recent reports suggested that *p*-type metal oxides may be more humidity-tolerant than *n*-type ones [127]. For instance, Drozdowska et al. [128] proposed nanoporous NiO films for NO_2_ detection. The NiO sensors exhibited stable response to NO_2_ at 150 °C under humid conditions (40% RH), indicating the less humidity-dependent characteristics of p-type metal oxides. Similarly, Wilson et al. [129] prepared ultrathin NiO films for gas sensing. Negligible drifts of baseline resistance were observed at a wide humidity range (0–70% RH) at 150 °C in NiO sensor (film thickness of 8 nm). Additionally, good repeatability was obtained when the NiO sensors were exposed to NO_2_ with staircase concentration (0.8–7 ppm) for two cyclic measurements at 50% RH. Miao et al. [130] reported one humidity-independent H_2_S gas sensor based on a monolayer film of CuO nanosheets, as shown in Figure 11g. The produced CuS_2_ and S during the reaction between hydroxyl groups and H_2_S on the surface of CuO under humid conditions served as the resistive surface layer to screen the humidity effect on gas sensing, as depicted in Figure 11h. It was found that the ultra-thin film structure was critical to achieve the humidity independence, by facilitating the diffusion of H_2_S in the shrinking pores and thus producing the Cu_x_S/S resistive layer. These results suggest that *p*-type metal oxides are promising materials to design humidity-tolerant sensors, which warrants more extensive investigation.

## 4. Challenges and Perspectives

In this review, recent progress in a series of anti-humidity strategies of CGS is summarized, including surface engineering, physical isolation, working parameters modulation, humidity compensation, and developing novel gas-sensing materials. Based on these anti-humidity strategies, recently reported studies have achieved inspiring humidity tolerance in gas detection. Despite the significant achievements, there are still some challenges to be overcome in the design of anti-humidity gas sensors in the future:

(I) As presented in this review, surface engineering is the most widely studied anti-humidity strategy. In regard to traditional gas-sensing materials that have been intensively investigated, direct surface engineering is one simple method, which mainly includes five forms of functionalization of noble metals, element doping, modification with hydrophobic materials, composites with hydrophilic materials, and post-treatment. The basic principle of these five surface engineering methods is introducing additives to functionalize gas-sensing materials. Therefore, the adsorption sites on gas-sensing materials are inevitably occupied, leading to response degradation. This phenomenon is prevalent for surface engineering-type gas sensors as demonstrated in Table 1. Meanwhile, we could find that the perfect compatibility was achieved between anti-humidity and sensitivity within some gas sensors in Table 1; namely, the additives should possess the dual function of boosting gas sensitivity and improving the humidity resistance. For instance, noble metals possess sensitization effects, the amino groups in APTES could selectively react with NO_2_ molecules, and heterojunctions could promote sensitivity. Therefore, surface engineering with dual functions should be considered preferentially.

(II) Utilizing semipermeable membranes or molecular sieves to isolate sensing film from water vapor could effectively promote the humidity tolerance of gas sensors. Whereas, the diffusion of target gases would be hindered by these membrane or molecular sieves to some extent, leading to a decline in sensitivity (Table 1). Furthermore, the sensitivity probably decreases with the elevating thickness of membranes or molecular sieves due to the permeability recession. The thickness of semipermeable membranes and molecular sieves should be optimized to obtain good anti-humidity performances with the least loss of sensitivity.

(III) In regard to CGS, sensitivity is closely related to the operating temperature. Although the elevation of operating temperature could improve the humidity tolerance of sensors, if the operating temperature does not match the optimal one, then the elevation of operating temperature for humidity tolerance probably would lead to a sensitivity recession. Light illumination has been intensively used to improve the sensitivity of gas sensors. Meanwhile, introducing light illumination to enhance humidity tolerance can be seen as one good way to kill two birds with one stone. However, in regard to working parameters modulation for anti-humidity performance, both the elevation of operating temperature and addition of illumination would increase the complexity of systems and energy consumption.

(IV) An overwhelming majority of studies in regard to anti-humidity gas sensor focused on minimizing the attenuation of sensor response to target gas within humid conditions. In fact, adsorbed water molecules may promote the reaction between adsorption sites on the sensing film and target gas molecules, and thus improve the sensor sensitivity. Humidity compensation based on mathematical models and neural network is one promising way to obtain the real concentration of target gas regardless of the negative or positive interferences from humidity. Of note, only one compensation model probably cannot fit in a wide humidity range, especially considering the physisorption of water molecules at high humidity. In regard to compensation based on the neural network, abundant data with gas-sensing features under different humidity conditions are essential to train the neural network. Therefore, sensors with high stability are very important to ensure constant sensing features for a long time.

(V) Developing novel gas-sensing materials with humidity tolerance renders the possibility to thoroughly eliminate humidity interference for gas sensors. The underappreciated *p*-type metal oxides in the past deserve more attention due to their humidity tolerance. Whereas, the process to develop new materials may be lengthy and uncertain. With the emerging of new technologies, machine learning and big data technology can theoretically design new materials before preparation, which can effectively shorten the time of developing new materials and propose various application-oriented solutions [131,132].

(VI) Almost all the previous reports in this review only utilized a single strategy to improve the humidity resistance of gas sensors. Resembling co-doping hydrophobic APTES and Ag nanoparticles to achieve the stable acetone sensing of Si nanowires [64], a combination of two or more anti-humidity methods probably further promotes the humidity tolerance of gas sensors.

In the process of summarizing the latest research advances of anti-humidity gas sensors, we realized that the studies in this field are still insufficient. Moreover, in regard to the emerging subject, many endeavors need to be devoted to overcoming these challenges in the development of anti-humidity gas sensors. Therefore, we hope this review will enlighten readers and open new avenues to further explore novel gas sensors with excellent anti-humidity features without other performance cost indexes.

## Figures and Tables

**Table 1:** The anti-humidity performances of recently-reported gas sensors.

Anti-Humidity Strategies			Target Gas and OT	Baseline Resistance Drift	Response Drift	Response Decline? *	Ref.
surface engineering	functionalization of noble metals	Pd-SnO_2_	H_2_ (600 ppm) 300 °C	-	-	Yes	[58]
		Au-Sn-ZnO	Benzene (49.4 ppm) 350 °C	-	-	No	[60]
		Ru-NiO	Acetone (100 ppm) 200 °C	-	3% (15–90% RH)	No	[63]
		Ag-APTES/Si nanowires	Acetone (1 ppm) RT	-	-	No	[64]
		Pt-SWCNTs	NO_2_ (1 ppm) RT	-	-	No	[65]
		Rh-ZnO	TMA (10 ppm) 180 °C		28.3% (55–90% RH)	No	[41]
	elements doping	Ce-In_2_O_3_	Acetone (10 ppm) 450 °C	3% (dry–80% RH)	5% (dry–80% RH)	Yes	[70]
		Ce-SnO_2_	NO_2_ (1 ppm) 140 °C	-	-	No	[42]
		Pr-In_2_O_3_	Acetone (20 ppm) 450 °C	~0% (0–80% RH)	~0% (0–80% RH)	Yes	[71]
		Pr-Co_3_O_4_	Acetone (50 ppm) 160 °C		8.82% (30–90% RH)	Yes	[72]
		Tb-SnO_2_	Acetone (20 ppm) 450 °C	23.3% (dry–80% RH)	20.1% (dry–80% RH)	Yes	[73]
		Pr-Ce-WO_3_	TMA (20 ppm) 300 °C	~0% (dry–80% RH)	~0% (dry–80% RH)	Yes	[74]
		Sb-SnO_2_	H_2_ (200 ppm) 350 °C	-	-	Yes	[75]
		Al-SnO_2_	Ethanol (100 ppm) 250 °C	-	-	Yes	[76]
	modification with hydrophobic materials	PDMS-Pd/TiO_2_	H_2_ (10000 ppm) 25 °C	20% (25–75% RH)	~0% (25–75% RH)	Yes	[83]
		PDMS-CoSnO_3_@MOF	NH_3_ (100 ppm) 160 °C	-	-	No	[25]
		APTES-WO_3_	NO_2_ (1 ppm) 340 °C	-	19.1% (25–90% RH)	No	[86]
		fluoroalkylsilane-modified BP	NO_2_ (1 ppm) 25 °C	-	-	No	[89]
		OTS-Si	NO_2_ (50 ppb) RT	-	19.3% (25–55% RH)	Yes	[90]
		PANI-MWCNTs	NH_3_ (100 ppb) 25 °C	-	19.6% (15–82% RH)	No	[84]
		PANI-Bi_2_MoO_6_	NH_3_ (1 ppb) RT		13.5% (40–90% RH)	No	[85]
		PVDF-PNDC	NO_2_ (1 ppm) RT	-	-	No	[91]
		Graphite-In_2_O_3_	NO_2_ (1 ppm) 75 °C	-	7% (20–90% RH)	No	[93]
		Graphite-PS-In_2_O_3_	H_2_S (100 ppb) RT	-	-	No	[94]
		MWCNT-WS_2_	NH_3_ (1 ppm) 16 °C	-	~0% (70–90% RH)	-	[95]
		C-WO_3_	H_2_S (100 ppm) 275 °C		10% (20–98% RH)	No	[96]
		SnS_2_-S/rGO	NO_2_ (1 ppm) RT	-	-	No	[97]
		ZrO_2_-SnO_2_	TEA (100 ppm) 190 °C	-	18% (50–90% RH)	No	[98]
		Y_2_O_3_-SnO_2_	NO_2_ (10 ppm) 200 °C		~0% (0–87% RH)	No	[99]
		CeO_2_-SnO_2_	TEA (20 ppm) 190 °C		14.1% (45–96% RH)	No	[100]
	composites with hydrophilic materials	NiO-SnO_2_	CO (50 ppm) 400 °C	~0% (dry–25% RH)	-	Yes	[101]
		NiO-NiFe_2_O_4_	acetone (100 ppm) 200 °C	60% (35–95% RH)	9.5% (35–95% RH)	-	[102]
		NiO-ZnO	NO_2_ (10 ppm) 350 °C		~0% (dry–81% RH)	No	[103]
		CuO-SnO_2_	H_2_S (1 ppm) 250 °C	-	-	No	[104]
		CuO-In_2_O_3_	CO (100 ppm) 200 °C	-	14.4% (25–95% RH)	No	[105]
		ACS-SnO_2_	NO_2_ (10 ppm) 150 °C	-	25% (dry–93.1% RH)	No	[43]
		SiO_2_-Cr_2_O_3_	H_2_S (5 ppm) 170 °C	9.9% (33–94% RH)	10% (33–94% RH)	-	[106]
	post-treatment	proton-beam irradiation- ZnO	NO_2_ (10 ppm) 300 °C		22.8% (0–75% RH)	No	[107]
		plasma fluorination-CNTs	NO_2_ (10 ppm) RT	-	-	No	[108]
		fluorocarbon plasma- In_2_O_3_	NO_2_ (1 ppm) 200 °C	-	~50% (6–92% RH)	Yes	[109]
		etching-SnO_2_/Zn_2_SnO_4_	H_2_O_2_ (1000 ppm) RT	-	-	No	[110]
physical isolation		PLA-CTAB/BP	CO_2_ (500 ppm) RT	-	-	Yes	[112]
		PDMS-Si	NO (5 ppm) RT	-	-	Yes	[44]
		PDMS-LIG	NO (1 ppm) RT	-	-	Yes	[115]
		3MCM-45-Au/SnO_2_	Formaldehyde (5 ppm) 110 °C		17% (32–91% RH)	No	[116]
		SBA-15-Pt/In_2_O_3_	Acetone (1 ppm) 320 °C	-	-	No	[117]
		PTFE-Mxene/TiO_2_	NH_3_ (1 ppm) 20 °C	-	-	-	[118]
		Al_2_O_3_-SnO_2_	NO_2_ (100 ppm) 300 °C	-	-	Yes	[45]
		Al_2_O_3_-Pt/ZnO	Acetylene (20 ppm) 120 °C	-	6% (dry–50% RH)	Yes	[119]
working parameters m modulation		Increasing OT- SnO_2_/RGOH	NO_2_ (3 ppm) 54 °C		9.9% (5–80% RH)	Yes	[46]
		UV illumination WS_2_/PbS	NO_2_ (1 ppm) RT	-	8.8% (10–90% RH)	No	[47]

* represents the comparison of response to target gas at dry conditions between anti-humidity gas sensors and untreated sensors.

## Data Availability

Not applicable.
